# The era of increasing cancer survivorship: Trends in fertility preservation, medico-legal implications, and ethical challenges

**DOI:** 10.1515/med-2025-1144

**Published:** 2025-02-12

**Authors:** Lina De Paola, Gabriele Napoletano, Giuseppe Gullo, Francesco Circosta, Gianluca Montanari Vergallo, Susanna Marinelli

**Affiliations:** Department of Anatomical, Histological, Forensic and Orthopedic Sciences, Sapienza University of Rome, Rome, Italy; Department of Obstetrics and Gynecology, Villa Sofia Cervello Hospital, University of Palermo, Palermo, 90146, Italy; Department of Clinical, Internal, Anesthesiological and Cardiovascular Sciences, “Sapienza” University of Rome, Rome, Italy; School of Law, Polytechnic University of Marche, 60121, Ancona, Italy

**Keywords:** oncology, fertility preservation, ethic/legal implication, cancer

## Abstract

**Introduction:**

Global cancer cases are increasing, but fortunately, cancer is becoming more treatable. By 2050, the number of cancer cases is projected to reach 35 million. These numbers are certainly correlated with the aging population, early diagnoses due to screenings, and the broad current treatment options. However, life-saving therapies are often gonadotoxic, significantly impacting the lives of cancer patients. Fertility preservation following life-saving oncological treatments is one of the challenges faced by patients with cancer.

**Material and method:**

We analyzed 73 articles to investigate the current state of fertility preservation in oncology, also evaluating the medico-legal implications.

**Results:**

The data indicate a growing trend of cancer recoveries and survivorship with opportunities to access fertility preservation through various methods, which are not entirely known or consistently offered to patients in the appropriate manner.

**Conclusions:**

The ethical and medico-legal aspects are numerous and seem to be still evolving.

## 1 Introduction

Global cancer cases are increasing, but fortunately, cancer is becoming more treatable. The report “The Numbers of Cancer 2023,” resulting from the collaboration between AIRTUM (Italian Association of Cancer Registries), AIOM (Italian Association of Medical Oncology), AIOM Foundation, and PASSI (Progress in Health Enterprises for Health in Italy), estimates that in 2023, cancer cases increased by over 18,000, reaching approximately 395,000 cases (208,000 in men and 187,000 in women) [1]. Despite these seemingly discouraging data on cancer incidence, the numbers for cancer treatments and survival rates are equally astounding. According to the Global Cancer Statistics 2024, it is estimated that in 2022, 20 million new cancer cases were diagnosed, and 9.7 million people died from the disease worldwide. By 2050, the number of cancer cases is projected to reach 35 million [2]. These numbers are certainly correlated with the aging population, early diagnoses due to screenings, and the broad range of currently available treatment options. Consequently, the focus has shifted from merely curing cancer to emphasizing survival and quality of life [3,4,5].

However, life-saving therapies are often gonadotoxic, significantly impacting the lives of patients with cancer. These patients face major psychological and physical stress, comparable to many psychiatric conditions or stressful situations [6], in addition to other challenges that complicate their journey [7]. Fertility preservation following life-saving oncological treatments is among such challenges since cancer patients often lack the knowledge and necessary information to make the decision that best meets their needs [8].

Our study aims to analyze the current state of oncofertility and describe potential innovations in fertility preservation within the oncological field. Additionally, it seeks to evaluate the possible social, ethical, and medico-legal implications of this highly sensitive topic. The ultimate goal is to foster debate and provide greater knowledge and awareness on the subject for both healthcare professionals and patients.

## 2 Materials and methods

**Figure 1:**
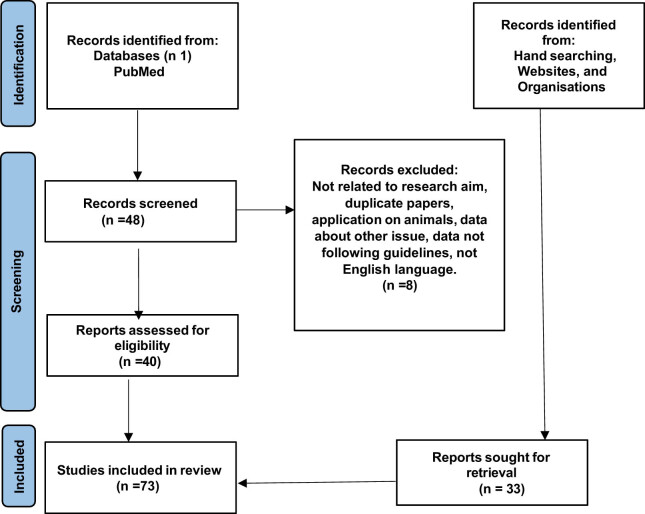
Prisma flow chart.

The present study analyzed articles available on PubMed over the past 10 years, from 2014 to 2024. Using the search terms “fertility” AND “preservation” AND “cancer” AND “legal,” 48 results were found. Articles identified through databases were screened by title and abstract. The full texts of potentially relevant articles were then analyzed and reviewed to assess their eligibility. Only articles written in English and addressing the topic of oncofertility were included. Duplicates and results unrelated to the subject matter were excluded. We included all types of papers, from case reports to reviews, without any exclusion criteria, to ensure our work was as comprehensive as possible. Cases involving both sexes (M/F) were considered. Ongoing studies were not included in the analysis. Additional data were incorporated into the study after manually searching websites and organizational documents to better understand the focus of the study. In particular, we searched for legal literature on the official institutional websites of the countries of interest, with a specific focus on Italy. Ultimately, as summarized in Figure 1, 73 articles were deemed suitable for inclusion in this study. This article aims to analyze the current state of oncofertility and potential innovations in fertility preservation in the oncological field, also evaluating ethical and medico-legal implications.

## 3 Results

Despite the high incidence rates of cancer, survival rates for oncological diseases are also increasing. Additionally, data indicate that cancers are unfortunately starting to appear at increasingly younger ages, with patients becoming younger. Although the incidence rate remains lower compared to older age groups, the socio-economic impacts are significant, particularly due to the loss of fertility. According to the study “Cancer incidence and mortality among young adults aged 20–39 years worldwide in 2012: a population-based study,” the increase in cancer survival rates implies that fertility can be jeopardized by the aggressive yet life-saving therapies administered. The types of cancers can vary significantly by age or geographic location, but the therapies are often gonadotoxic. Consequently, both men and women, who have not yet fulfilled or have partially fulfilled their desire to procreate, face the threat of infertility due to the disease or its treatment [9].

In this context, the field of oncofertility emerges, which is a branch of oncology intersecting with reproductive medicine. Its goal is to preserve the reproductive reserve of cancer survivors [4,10]. International guidelines indicate that oncologists have a duty to adequately inform patients with cancer about the potential infertility associated with gonadotoxic treatment and to inform them about the possibility of using fertility preservation methods by referring them to reproductive medicine pathways [11]. Reproductive counseling for young cancer patients should be proposed immediately after the diagnosis and subsequent staging of the oncological disease, to allow sufficient time to share the best fertility preservation strategies, which vary according to the oncological and reproductive prognosis. During the initial oncological assessments, it is recommended to discuss the best fertility preservation strategies that vary based on the oncological and reproductive prognosis. Counseling requires a multidisciplinary approach and effective communication between the oncologist and the reproductive medicine specialist [12]. During counseling, the patient’s interest in future pregnancy, motherhood, or fatherhood should be explored [13]. The risk of infertility from the proposed treatment and the oncological prognosis should be estimated. If the patient is interested and indicated, the various methods of gamete preservation or reduction of gonadal toxicity available should be explained [14].

Oncofertility involves various professionals working in a team, including oncologists, gynecologists specializing in reproductive medicine, and experts in social sciences, utilizing diverse methods [15,16,17,18,19,20]. Currently, there are several options for fertility preservation in patients with cancer, and the most advanced techniques have significantly improved their quality of life. In the last 10 years, among the most advanced methods, cryopreservation is noteworthy, involving the retrieval and subsequent freezing of oocytes or embryos before the initiation of oncological treatment. Additionally, the use of gonadotropin-releasing hormone agonists and antagonists has further improved these techniques by protecting ovarian function during chemotherapy. Studies have shown that these procedures help preserve fertility without compromising the success or outcome of cancer treatment [21,22]. Other promising developments include *in vitro* maturation of oocytes, which allows for the maturation of oocytes outside the body, and the potential use of an artificial ovary, which could minimize, albeit slightly, the risk of reintroducing cancerous cells harvested during fertility preservation procedures into a healthy body. Although some of these techniques are still under investigation, they represent a promising scenario for even safer fertility preservation options [22]. Furthermore, research indicates that fertility preservation does not increase the risks of cancer recurrence nor negatively impact the health of children born to cancer survivors. These advancements provide reassurance from both a strictly medical and psychological perspective to patients considering fertility preservation during their cancer treatment journey [22,23,24].

Despite the increasing dissemination of these techniques, studies published in the literature indicate that the issue of fertility is not always adequately addressed in cancer patients, depriving them of the opportunity to access effective methods and procedures. Among the primary reasons for patients not adhering to fertility preservation programs are the need to start chemotherapy treatment as soon as possible, the costs of the procedures [25], lack of clarity during discussions with the doctor (due to an overload of information or difficulty in asking for clarifications later), and insufficient psychological support. Regarding the difficulties encountered by medical staff, there is an incomplete knowledge of the topic, insufficient resources provided by the facilities where they work, and a lack of organization with a specialized team.

From a surgical perspective, it is essential to highlight the critical role of minimally invasive procedures in the context of oncofertility. For lesions localized within the reproductive system, certain techniques can effectively minimize damage to the structural integrity of the organs while preserving fertility. These procedures are vital for safeguarding reproductive potential without compromising oncological safety, making them a key option for young women seeking to balance effective cancer treatment with fertility preservation [26]. Therefore, fertility preservation is implemented both in cases where the reproductive organs are directly affected by a neoplastic disease and when the disease pertains to another organ system, but the antineoplastic therapy is highly or potentially harmful to the patient’s fertility. Among fertility preservation methods, the most widely used is cryopreservation, involving the freezing of gonadal tissue [27,28,29].

Beyond the oncological problem, patients are subjected to significant psychological stress. Many studies highlight how cancer represents a substantial stress factor that can trigger underlying psychiatric conditions, form the basis of psychological disorders, and heavily impact the psychosocial sphere of the survivor. The distress derived from a new self-conception, along with associated fears for one’s future life, can persist for a long time, particularly if the neoplasm occurs at a young age. Cancer can pose a long-term risk for developing psychiatric disorders and, for example, has been identified as a risk factor for the development of PTSD [30]. Like other stressful events, psychological support throughout the entire therapeutic and preventive treatment process for infertility must always be considered [31].

A highly debated medico-legal issue when treating patients with cancer concerns the possibility that preparing for fertility preservation might increase the risk of tumor cell dissemination, thereby worsening the prognosis. This dilemma highlights the conflict between the patient’s right to self-determination and the physician’s principle of non-maleficence [32,33]. To date, no cases have been described in which actual worsening occurred due to hormonal stimulation (for gamete collection) or the postponement of chemotherapy treatment. However, this is a risk that must be considered when proposing these procedures, keeping in mind that treatments must be as personalized as possible [34,35,36,37,38,39]. Another risk lies in the possibility that the harvested and cryopreserved tissue may already contain tumor cells, raising the danger that during reimplantation, facilitated by hormonal hyperstimulation, there could be a resurgence of neoplastic disease. In essence, the physician proposing a fertility preservation treatment must explain to a patient, already facing a dire diagnosis, that there is hope for future parenthood but that the exact health risks involved cannot be fully determined. This places a burden on the physician, who must balance the obligation to provide the patient with the best possible information for making an informed choice with the duty to safeguard the patient’s health as a primary concern [40,41,42].

Another point of reflection concerns the mistaken belief held by some cancer patients and healthcare providers that children born to a cancer survivor may have a higher risk of developing cancer. This hypothesis is currently unsupported by any data in the literature and therefore cannot be used as a justification for not proposing any fertility preservation treatment [43,44,45]. A second aspect involves the scenario where the cancer survivor’s parent dies prematurely, leaving the child orphaned. However, this concern is also insufficient to justify denying the opportunity for fertility preservation [46,47,48]. This issue becomes even more controversial in cases where patients are single at the time of diagnosis [48,49,50]. Another ethical issue concerns the use of gametes after the donor’s death or a change of mind by the individual who donated them [51,52]. Internationally, there are various positions on this matter [53]. In Italy, assisted reproduction procedures are regulated by Law 40 of 2004. However, new guidelines published in recent months clarify some measures of the law and replace those from 2015. It is specified that after assisted fertilization of the egg, consent to medically assisted procreation (MAP) cannot be revoked. Therefore, the woman can request the implantation of the embryo even in the event of the partner’s death or separation. The guidelines state: “It must be noted that after the assisted fertilization of the egg, consent to MAP cannot be revoked, and the woman can request the implantation of the embryo even if the partner has died (Cass., 15 May 2019, no. 13000) or if their relationship has ended (Constitutional Court, no. 161/2023).”

The guidelines also emphasize that access to MAP is extended to fertile couples carrying transmissible genetic diseases, as well as to serodiscordant couples with infectious diseases such as HIV, HBV, and HCV, where the high risk of infection effectively constitutes a barrier to procreation; and to couples where one or both partners have previously undergone cryopreservation of their gametes or gonadal tissue for fertility preservation. These clarifications finally address some of the ambiguities faced by oncological patients and their partners.

Ovarian tissue cryopreservation (OTC) is a significant technique for preserving fertility, particularly for prepubescent girls and adolescents affected by cancer [54,55]. However, its use raises various ethical, legal, and social concerns. For younger girls, the primary ethical issue is their inability to make autonomous decisions due to their legal and cognitive immaturity. Parents must make decisions on their behalf, raising ethical concerns about whether these choices truly reflect the child’s needs and future preferences. For adolescents, the situation becomes even more complex: while they are informed and involved in the decision-making process alongside their parents, they may not fully understand the implications of a choice that could profoundly impact their future [56].

Moreover, OTC requires a surgical procedure that carries general risks associated with surgery and specific risks related to the technique itself. Although it was considered an experimental technique until recently, recent evidence and updated guidelines highlight the necessity of recognizing OTC as an integral part of fertility preservation treatment options. Nonetheless, access to this procedure often remains challenging, particularly as it frequently depends on a family’s financial resources, thereby creating inequalities in the exercise of the right to health and self-determination [57]. From a legal perspective, many gray areas surround OTC. Until recently, this technique was considered a non-standard procedure for prepubescent girls, potentially causing confusion among families who may not fully understand that it is now recognized by recent guidelines as a reliable fertility preservation technique. The number of live births resulting from cryopreserved ovarian tissue continues to increase globally. Nevertheless, the lack of clarity could lead to potential legal disputes, imposing burdens on both patients and healthcare institutions [58].

Another critical legal question concerns ownership of the cryopreserved tissue. Who owns the tissue once it is collected? What happens if, upon reaching adulthood, the individual decides not to use it or is unable to do so? Current regulations often lack clear guidelines on ownership, storage, and associated costs, which can become a long-term financial burden [53]. In Italy, unused cryopreserved tissue is stored indefinitely in specialized “banks.” It can also be donated or discarded with a written request, as it is considered biological material rather than embryos, which, by law, must be preserved indefinitely. If OTC is performed for oncological reasons, the procedure is free of charge; in other cases, costs are defined by the clinics providing the treatment. On a social level, parents of minors affected by cancer often feel compelled to do everything possible to preserve their child’s fertility, even when the likelihood of achieving the desired outcome is unclear. Cultural factors may also play a role, as fertility carries significant symbolic value in certain societies, potentially influencing decisions and the girl’s future life path. From a psychological perspective, adolescents who are aware of their fertility issues may experience considerable emotional stress, particularly if the technique does not work as hoped. This can lead to psychological challenges that are difficult to manage and that require ongoing support [5,13,53]. In the context of oncofertility, another dilemma arises: OTC can delay the initiation of cancer treatments, which often take precedence over fertility preservation. Additionally, the ovarian tissue obtained may be insufficient or of inadequate quality for future use due to the effects of the disease or treatments received. The role of healthcare professionals is therefore critical in providing thorough and tailored explanations to ensure genuinely informed consent. The interdisciplinary team must balance the urgency of cancer treatment with the desire to preserve fertility and provide adequate psychological support to the patient and her family. In summary, OTC offers great promise but requires careful management. Continuous dialogue between healthcare providers, families, and patients is essential to evaluate whether it is the right choice on a case-by-case basis. Decisions must be based on clear, current, and individualized information. Psychological support is crucial, and clear regulations are necessary to protect both the individuals involved and the healthcare institutions, minimizing unnecessary legal disputes.

## 4 Discussion

Cancer survivors are undoubtedly increasing, and due to the lower and lower age of diagnosis, younger patients constitute a significant portion of the survivor population. This implies an increase in cancer-free years, an increase in the number of years affected by cancer, and a reduction in fertility. The social burden of this situation is not negligible. Undergoing fertility preservation treatments adds to the therapies a cancer patient must undergo. The increase in medical therapies translates into a significant economic and social cost, which burdens the state budget in countries with public healthcare systems, such as Italy [59].

In fact, regarding the ethical aspects of fertility preservation in cancer patients, several key considerations emerge. The first concerns informed consent and autonomy, whereby cancer patients, particularly those of reproductive age, are entitled to be made aware of the infertility risk arising from gonadotoxic forms of treatment. Physicians are ethically bound to provide thorough information, in an intelligible fashion, as to fertility preservation options, to enable patients to make fully informed decisions. This is particularly relevant for young patients, who are in the most fertile period of their lives and may not fully understand the long-term impact of their choices due to the stress of their diagnosis. Informed consent entails even greater complexities in cases involving minors, raising questions about parental involvement and the patient’s own consent [23]. Another ethically relevant issue is the availability and accessibility of fertility preservation techniques. Patients may not only be inadequately informed but also lack the financial means to undergo oocyte or embryo cryopreservation, which could lead to significant disparities in care. Such disparities already exist, particularly for patients in low-income regions, who face greater barriers. Ethical principles of justice therefore require healthcare professionals to strive for equitable access and offer these options consistently to all eligible patients [23]. A final ethical aspect concerns the timing of fertility preservation treatments and the balance of risk and benefit. The timing required for proper fertility preservation may delay life-saving cancer treatments. While the preservation of fertility is important for patients who wish to have children, it must be balanced against the urgency of starting oncological treatment. This creates a complex risk-benefit analysis, as even a slight delay in treatment could negatively impact the cancer prognosis, raising ethical issues between preserving future fertility and maximizing immediate survival [22].

These ethical considerations naturally carry medico-legal implications. Physicians may face legal challenges if they fail to inform patients about fertility preservation or if preservation procedures result in unexpected complications, according to industry guidelines and best medical practices. [11,14,60,61]. Legal frameworks must balance the principle of non-maleficence – *non-nocere* – inherent in medical practice with the patient’s right to autonomy and self-determination, especially in cases where procedures carry minimal but real risks [22,23]. Moreover, the psychological burden faced by the individual can lead to traumas that are not always easily overcome without professional help, often requiring psychological therapies with additional costs for the survivor. Finally, beyond the direct impact, there is also a social burden that the survivor must bear alone. This is exemplified by the denial of the right to be forgotten regarding cancer. Patients often find themselves battling administrative and bureaucratic paperwork for decades, which denies them some of the most basic rights of civil society [7,62,63].

Finally, new technologies such as artificial intelligence (AI) have already significantly increased survival rates and improved the quality of life for patients with cancer. AI could potentially be used even more extensively, not only for diagnostic methods but possibly also for predictive functions. It can, for example, be used for the selection of oocytes or sperm, or for the evaluation of *in vitro* fertilization, including embryo assessment. It could also be useful for micromanipulation procedures, such as intracytoplasmic sperm injection into the oocyte. In short, AI would be an excellent tool to use, but the main limitation currently seems to be the establishment of high-quality databases. Identifying who would bear responsibility for any potential harm to individuals remains a challenge [64,65,66,67,68,69,70]. Another question to consider is the duration of storage for reproductive material. A young patient might not wish to become a parent for many years, and thus, the maintenance of gametic material for future procedures also represents a societal cost [71,72,73,74]. Therefore, one must also consider whether and for how long ethical considerations and the value assigned to reproductive material should outweigh the current social and economic costs of such procedures.

In summary, the ethical aspects of fertility preservation in cancer patients involve ensuring informed consent, equitable access, careful consideration of timing and risks, and managing the evolving medico-legal landscape. These considerations are crucial for the holistic care of cancer survivors.

## 5 Conclusions

The conducted study has revealed a global picture with discrepancies between various countries regarding the possibility of fertility preservation. These differences are supported by cultural, religious, and economic factors, in addition to scientific advancements and the availability of innovative treatments. Obstacles include high economic costs, inadequate doctor–patient communication, insufficient knowledge among non-specialist healthcare providers, subjective fears of the patient regarding potential offspring and their prognosis, concerns about losing certain rights, and uncertainty about the duration of gamete storage. However, internationally, there has been increasing attention to this issue in recent years due to the growing number of cancer survivors and their younger age. The situation appears to be continuously evolving and will require further research and updates in the coming years. Thus, greater awareness and consistency in offering these options are essential to further improve the quality of life of cancer survivors.

## 6 Limitations

Our study presents clear limitations. The research is primarily based on data collected from PubMed and a limited selection of keywords, which may have excluded relevant articles on the topic. The choice to consider only the last 10 years in our research is limiting, but it was made considering that the topic has undergone significant changes, particularly in this last decade. Additionally, we discussed the role of emerging technologies, such as AI; however, we were unable to thoroughly explore their broader implications, such as costs, accessibility, and ethical concerns. These limitations, however, represent a starting point for future research that can expand upon and address the broader issues raised.
